# The Current Landscape of Secondary Malignancies after CAR T-Cell Therapies: How Could Malignancies Be Prevented?

**DOI:** 10.3390/ijms25179518

**Published:** 2024-09-01

**Authors:** Stella Bouziana, Dimitrios Bouzianas

**Affiliations:** 1 Department of Hematology, King’s College Hospital, London SE59RS, UK; 2 BReMeL, Biopharmaceutical and Regenerative Medicine Laboratories, 55534 Thessaloniki, Greece; dimbou55@gmail.com

**Keywords:** CAR T-cell therapies, relapsed/refractory haematological malignancies, secondary T-cell lymphomas/leukaemias, secondary malignancies, viral vectors, genome editing, genotoxicity

## Abstract

Chimeric antigen receptor (CAR) T-cell therapies have revolutionised the field of haematological malignancies by achieving impressive remission rates in patients with highly refractory haematological malignancies, improving overall survival. To date, six commercial anti-CD19 and anti-BCMA CAR T-cell products have been approved by the Food and Drug Administration (FDA) for the treatment of relapsed/refractory B-cell haematological malignancies and multiple myeloma. The indications for CAR T-cell therapies are gradually expanding, with these therapies being investigated in a variety of diseases, including non-malignant ones. Despite the great success, there are several challenges surrounding CAR T-cell therapies, such as non-durable responses and high-grade toxicities. In addition, a new safety concern was added by the FDA on 28 November 2023 following reports of T-cell malignancies in patients previously treated with either anti-CD19 or anti-BCMA autologous CAR T-cell therapies both in clinical trials and in the real-world setting. Since then, several reports have been published presenting the incidence and analysing the risks of other secondary malignancies after CAR T-cell therapies. In this opinion article, the current landscape of secondary malignancies after CAR T-cell therapies is presented, along with a proposed strategy for future research aiming at potentially diminishing or abrogating the risk of developing secondary malignancies after CAR T-cell therapies.

## 1. Introduction: Current CAR T-Cell Treatment Landscape

The broad application of novel technologies and the advancement of genetic engineering have resulted in the development of chimeric antigen receptor (CAR) T-cell therapies, which have revolutionised the field of haematological malignancies. CAR T-cell therapies are engineered to selectively target and eliminate tumour cells while recruiting and activating host immune cells as scavengers in the battle against the killing of cancer cells [1]. T-cells are collected through leukapheresis, and a synthetic transgene is incorporated into the genome of T-cells ex vivo by a replication-defective self-inactivated viral vector to express a CAR on the surface of T-cells [2]. CARs comprise an antigen-binding extracellular immunoglobulin-derived single-chain variable fragment domain fused to a hinge and a transmembrane region. The latter binds to an intracellular costimulatory domain in second-generation products, either 4-1BB or CD28, followed by the CD3z T-cell receptor signalling chain. Currently, there are five generations of CAR T-cells under development [3,4]. CAR T-cells are infused after the administration of a lymphodepleting regimen that creates a favourable immune environment for CAR T-cells to proliferate and function. Once bound to the targeted antigen, CAR T-cells are activated and expanded in vivo to destroy cancer cells [3,5].

CAR T-cell therapies have proven extremely potent in inducing remission in patients with highly refractory and aggressive haematological malignancies, offering effective disease control and improving overall survival [6,7,8]. To date, six commercial anti-CD19 and anti-BCMA CAR T-cell products have been approved by the Food and Drug Administration (FDA) with the indication of treating relapsed/refractory (R/R) high-grade and indolent B-cell non-Hodgkin lymphomas (B-NHLs), B-cell acute lymphoblastic leukaemia (B-ALL) and multiple myeloma [9]. Recently, the FDA granted accelerated approval to one of these products (lisocabtagene maraleucel) for the treatment of adult patients with R/R chronic lymphocytic leukaemia or small lymphocytic lymphoma [10] (Table 1).

Despite the startling initial disease responses, long-term durable remissions are seen only in a minority of CAR T-treated patients, although there are a few reported cases considered cured [6,11]. Disease recurrence is one of the major obstacles in the field of CAR T-cell therapies [12]. In order to achieve prolonged remissions, different scientific groups try to develop more efficacious products by exploiting the principles of genetic engineering or other sophisticated technologies [13,14,15]. In addition, several other challenges surround the administration of CAR T-cell therapies, including high-grade toxicities and life-threating infections that may lead to prolonged hospitalisations, increase the financial burden for health care and insurance systems and impair patients’ quality of life or even lead to death [16,17].

Currently, there are no approved CAR T-cell products against solid tumours; however, multiple research teams across the globe are investigating the treatment potential of CAR T-cells in solid tumours, joining their forces to augment CAR T-cell efficacy in lethal and untreatable cancers [14,18]. In parallel, the potential of CAR T-cell therapies has been expanded beyond oncology in treating autoimmune diseases. Clinical data of the first-treated patients with a variety of autoimmune diseases is very impressive and promising [19,20]. Preclinical data deriving from other non-malignant conditions show the potential of CAR T-cells to change the treatment arena of a plethora of conditions, including chronic infections, cardiac fibrosis and senescence-associated diseases [21].

The potential of a broader applicability of CAR T-cell therapies has caused a lot of excitement. However, on 28 November 2023 the FDA announced reported cases of T-cell malignancies in patients previously treated with either anti-CD19- or anti-BCMA-directed autologous CAR T-cell therapies both in clinical trials and in the real-world setting, raising concerns about the safety of these promising therapies. The reports of T-cell malignancies were associated with serious outcomes, including hospitalisation and death [22]. In this opinion article, the current landscape of secondary malignancies after CAR T-cell therapies is presented, along with the risk of genotoxicity carried by gene delivery systems. In addition, a strategy for future research is proposed, aiming at potentially diminishing or abrogating the risk of developing secondary malignancies after CAR T-cell therapies.

## 2. Reports of Secondary Malignancies after CAR T-Cell Therapies and Potential Mechanistic Causes

In November 2023, the FDA released a statement regarding reports of malignancies of T-cell origin, both lymphomas and leukaemias, including CAR-positive cases, in patients who had been previously treated with either anti-CD19- or anti-BCMA-directed autologous CAR T-cell products. These reports derived from either clinical trials or post-marketing adverse event databases. The FDA has defined that the risk of T-cell malignancies concerns all the CAR T-cell products currently approved for commercial usage [22]. So far, 22 cases of secondary T-cell malignancies have been reported, with the majority (14 of 22 cases) occurring within 2 years after CAR T-cell administration (range 1 to 19 months) and one-third of cases manifesting in the first year. Further details on these cases are under investigation [23]. As more data are accumulated and more patients are treated with CAR T-cells under growing indications, this number may further increase. It is believed that the number of these reported cases may be an underestimation of the true incidence, as currently more than 34,400 patients have been treated with commercial CAR T-cell therapies and only 8000 cases have officially been reported in the FDA Adverse Events Reporting System (FAERS) database [24]. It is noteworthy that 8 of the 17 reported secondary T-cell lymphomas after CAR T therapy in the FAERS database reported death; it was unclear whether these patients died from the primary disease or the secondary T-cell lymphoma, as the FAERS reporting system does not allow the establishment of causal relationships [25]. The genetically modified CAR T-cell products, which have been recently introduced into clinical care, have been approved with the mandatory requirement of following up treated patients for 15 years in a post-authorisation safety framework to monitor for long-term adverse events, including the possibility of genotoxicity. Since November 2023, the FDA has announced that the safety observation for future malignancies will extend to a life-long requirement, whilst adding class-wide boxed warnings to all CAR T-cell therapies, which represents the highest safety-related caution for medical treatments [26].

The published data concerning these rare cases reported to the FDA are scant, and the exact mechanistic cause driving the development of secondary T-cell malignancies is unclear. So far, data derive from published anecdotal cases and retrospective cohorts. Importantly, of the 22 reported cases of T-cell malignancies, the CAR transgene has been identified in the malignant clones by genetic sequencing in only 3 of these cases [23]. The CAR transgene has been identified within the 3′ untranslated region of PBX2, a gene related to the development of lymphomas, in a secondary T-cell lymphoma case previously treated with ciltacabtagene autoleucel, a commercial anti-BCMA product. It was unclear whether the CAR integration contributed to the T-cell malignant transformation, because genetic mutations within the T-cells predated the CAR T-cell manufacturing and infusion [27]. It is believed that the disruption either of tumour suppressor genes or the activation of protooncogenes or other crucial genetic loci orchestrating transcription, translation regulation or cell activation could be the leading cause of lymphomagenesis [28]. Pre-existing T-cell clones with a malignant potential used for CAR T-cell manufacturing may be another contributing mechanism to oncogenesis. In these cases, the CAR transgene will inevitably be identified within the tumour cells and biopsies [27,29]. However, the number of CAR transgene copies identified in the tumour biopsies plays a significant role in interpreting the results, as very low levels may represent infiltrating CAR T-cells rather than the neoplastic T-cells, reflecting a case of a CAR-negative T-cell malignancy [29]. It is also believed that the continuous and prolonged stimulation of T-cells through the CAR or endogenous T-cell receptor may lead to secondary mutational events and progression to overt malignancies, serving as an additional driving mechanism [30].

**Figure 1:**
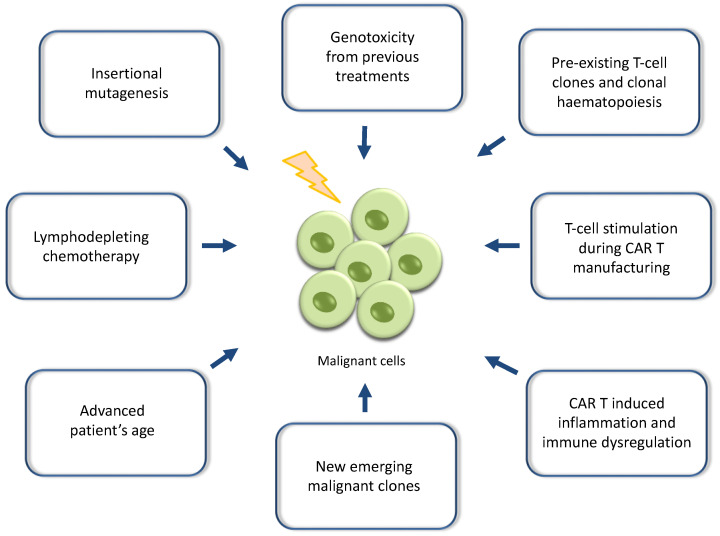
Potential mechanisms contributing to the development of secondary malignancies after CAR T-cell therapies: The exact mechanisms driving the occurrence of secondary malignancies, including T-cell malignancies after treatment with CAR T-cell therapies, are not well understood and remain a field under investigation. It is believed that these malignancies represent the result of multiple hits or combinatorial causative factors culminating in the emergence of malignant clones and overt oncogenesis.

In other cases, the CAR transgene has not been identified within the tumour cells, and secondary T-cell malignancies have not been associated with the CAR T-cell treatment as a causative factor [31,32,33]. Interestingly, patients with B-NHLs have a fivefold higher risk than the general healthy population of developing secondary T-cell lymphomas [34]. Similarly, secondary T-cell lymphomas have been reported after treatment with immune checkpoint inhibitors with a low incidence of 0.02%, while the rate currently reported for CAR T-cell therapies reaches up to 0.06% (22 out of 34,400 cases) [24]. It has been found that PD-1 has an additional role of being a tumour suppressor, and loss of PD-1 tumour suppressor function can lead to T-cell lymphomagenesis [35]. It is possible that the combination of previous multiple lines of treatment with chemoimmunotherapy, radiation or even autologous or allogeneic transplant, along with the immune dysregulation and inflammation induced by CAR T-cells, may drive the occurrence of CAR-negative T-cell malignancies as a multi-hit mechanism. These CAR-negative malignancies may represent the evolution of new emerging T-cell clones or pre-existing malignant clones that failed CAR transduction, and they were further stimulated by T-cell activation during CAR T-cell manufacturing and the subsequent inflammation caused by post-infusion CAR T-cells [29,33] (Figure 1).

Since the FDA announcement of the secondary T-cell malignancies after CAR T-cell therapy, a handful of reports have been published. They present the incidence of other secondary malignancies occurring after CAR T-cell therapies, as reported in the FAERS database or captured by the Center for International Blood and Marrow Transplant Research (CIBMTR) or by retrospective cohorts. Of note, most of the pivotal CAR T clinical trials also reported the incidence of secondary malignancies [25,36,37]. The relevant incidence reported in the FAERS database is 4.3% (536 of 12,394) out of all adverse event reports following CAR T-cell therapies, with axicabtagene ciloleucel and tisagenlecleucel comprising most of the secondary malignancy reports (51.7% and 33.0%, respectively) [25]. Real-world data report an incidence of secondary malignancies after commercial CAR T-cells ranging from 2.2% to 4.5%, varying between paediatric and adult reports and not being higher than expected in this population of patients previously exposed to cytotoxic therapies [25,29,38,39]. These neoplasms refer mainly to haematological myeloid malignancies, but they also include solid tumours, with a median time of presentation from CAR T infusion being 9 months [24]. The first category predominantly comprises myelodysplastic syndromes (MDS) and acute myeloid leukaemia. According to the FAERS database, the corresponding incidence is 38.8% and 19.8% out of all reported secondary malignancies [25]. Clinical trials with long-term follow-up have reported an incidence of secondary myeloid malignancies ranging from 4 to 16% of CAR T-treated patients [36,37,40]. Among the solid tumours, the most common are non-melanomatous skin cancers (7.8% of all reported secondary neoplasms in the FAERS database) [25]. A retrospective study performed by the University of Pennsylvania estimated that the 5-year incidence of secondary malignancies was 15.2% for solid cancers and 2.3% for haematological cancers [29]. Another single-centre retrospective study identified 25 cases of secondary solid cancers, excluding non-melanoma skin cancers, and 14 secondary haematological cancers, including 1 case of T-cell lymphoma, out of 724 patients treated with CAR T-cell therapies for either haematological malignancies or solid tumours who had a median follow-up of 15 months [33].

Multifactorial drivers are believed to play a role in the development of such secondary malignancies, especially the myeloid ones, including advanced age and the pre-existence of clonal haematopoiesis before CAR T-cell treatment potentially resulting from the exposure to previous intensive treatments. Indeed, a high proportion of patients ranging from 20% to 60% have been found to have clonal haematopoiesis of indeterminate potential at baseline before receiving CAR T-cells [41,42]. In addition, the CAR T-related inflammation developing after the infusion and onwards, especially by the CAR T-cells residing in the bone marrow, may accelerate the progression of abnormal haematopoietic clones to malignancies or the generation of new mutated clones in heavily pre-treated patients [43,44,45,46,47]. The lymphodepleting chemotherapy before CAR T may also play a contributing role by either suppressing a crucial subset of the cellular antitumour immunity or causing genotoxicity [48] (Figure 1).

Despite these reports, FDA has not pulled CAR T-cell therapies off the market, as the reported rate and risk of secondary malignancies, particularly T-cell malignancies, appears low compared with other cancer treatments. In addition, investigations on the driving mechanisms are still underway, and researchers are waiting for emerging data to enlighten the field to reach a consensus [23,24]. The true risk of potential insertional mutagenesis remains unknown given the high disease refractoriness of patients receiving CAR T-cell therapies. In addition, the principle of immortal time bias should also be taken into account, as patients receiving CAR T therapies currently achieve longer survival, which may translate to a higher cumulative risk of secondary malignancies in the background of R/R disease [49]. However, reports of T-cell and other malignancies after CAR T-cell therapy should prompt continued long-term investigations [22]. In alignment with the FDA, leading global organisations such as the American Society for Transplantation and Cellular Therapy, the European Society for Blood and Marrow Transplantation, the International Society for Cell and Gene Therapy and the CIBMTR recommend that CAR T-cells should be continued to be administered, as currently the benefits far outweigh the small and uncertain risks, which are much lower compared with other established cancer treatments [24]. However, strict surveillance and safety reporting to regulatory authorities are highly recommended, in addition to health care professionals being alert while monitoring CAR T-treated patients [22,24]. In the meantime, different research groups are trying to validate the significance of models incorporating established scoring systems, such as the CAR-HEMATOTOX and the Clonal Haematopoiesis Risk Score, in predicting the risk of developing secondary myeloid malignancies after CAR T-cell therapy and stratifying patients according to the relevant risk. This clinical stratification seems necessary in order to identify a subgroup of high-risk patients who will benefit most from closer surveillance after CAR T-cell therapy and potential additional screening before CAR T treatment [50,51].

## 3. Viral Vectors and Genetic Engineering Technologies in Gene Therapy and CAR T-Cell Manufacturing

The use of viruses as vectors in transferring transgenes has been the mainstay of transduction methods in gene therapy. The ability of certain viruses to integrate transgenes into the genome has been exploited in CAR T-cell manufacturing technology [52]. Vector integration into the host genome has always been a matter of concern in terms of the potential alteration of gene expression or the promotion of neoplastic transformation. Mechanisms include vector insertion into the gene enhancer or the promoter mediating gene overexpression or integration causing gene inactivation [53]. Currently, lentiviruses (LVs) and retroviruses constitute the most commonly used viral vectors in gene therapy. In the past, conventional retro-gamma viruses and adenoviruses were used as delivery machines in gene therapy. However, the initial triumph was overshadowed by reports of insertional mutagenesis resulting in leukaemia and MDS in patients treated with gene therapy using retro-gamma viruses for X-linked severe combined immunodeficiency, chronic granulomatous disease and Wiskott–Aldrich syndrome [54,55,56]. On the other hand, adenoviruses are strongly immunogenic, and they caused undesired immune responses in treated subjects [57]. Since then, viral vector technology has been massively refined to develop safer next-generation non-replication-competent viral vectors, including self-inactivating (SIN) vectors, enhancer-blocking insulators and microRNA targeting of vectors. There are multiple factors influencing vector-mediated genotoxicity, such as virus type, integration target site and target cell type, while non-viral factors such as patient age, disease type and dose are equally important. [58,59].

SIN LVs are currently preferably utilised, as they permit long-term transgene expression, have high packaging capacity and are deemed safer in integrating transgenes in specific genetic loci into the genome, confining random integration [53,60]. In addition, they have the advantage of enabling efficient transduction of nondividing or slowly dividing cells, surpassing the need for extra activating of the host cells [58]. On the downside, production with LVs is more costly, lengthy and cumbersome. However, SIN LV vectors may still be genotoxic, as despite integrating in a semirandom fashion, they tend toward genomic areas with active gene expression, including cancer-related genes [60]. Both in vitro and in vivo preclinical studies have shown that SIN LV integration caused aberrant splicing and premature termination of transcripts and tumour acceleration by disruption of tumour suppression genes or oncogene activation [61,62]. Notably, although initial concerns regarding the risk of potential insertional mutagenesis were regarded as more relevant in the transduction of hematopoietic stem cells, the recent FDA report on T-cell malignancies brings to light the risk of insertional mutagenesis in differentiated cells [63].

Although the FDA has not related any of the recently reported cases to vector-mediated oncogenicity so far, previously published cases have highlighted the possibility of clonal CAR T-cell expansion in anti-CD19 and anti-CD22 CAR T-cell therapies. This occurred via the integration of an LV either in the TET2 gene, which regulates haematopoiesis and T-cell differentiation, or in the CBL gene, which regulates T-cell responses [64,65]. These cases did not result in the development of neoplasms but demonstrate the risk of insertional mutagenesis. Of note, the clonal expansion of a myeloid-biased cell clone has also been reported in an adult patient with severe β-thalassaemia treated with LV β-globin gene transfer, in whom the integrated vector caused enhancer-mediated transcriptional activation of the HMGA2 gene with increased expression of a truncated HMGA2 mRNA insensitive to degradation [66]. In a clinical trial investigating hematopoietic stem-cell gene therapy for cerebral adrenoleukodystrophy, the top abundant integration sites of SIN LVs were genes involved in cell cycle regulation, cell division and oncogenesis; such genes included SGM6, CCND2 and MECOM, leading to clonal expansion and subsequent reports of MDS cases related to the viral insertion [67,68]. Rare cases of myeloid malignancies have been reported after hematopoietic stem-cell gene therapy in sickle cell disease. However, it is likely that the myeloablative conditioning chemotherapy previously administered resulted in the development of driver mutations associated with myeloid neoplasms or created positive selection pressure resulting in the progression of a pre-existing clone. It is well known that sickle cell patients harbour an inherently higher risk of developing clonal haematopoiesis and malignancies compared to the general population [69,70,71]. None of these cases have been explicitly related to random insertional mutagenesis caused by the viral vector [70,71].

Adeno-associated viruses (AAVs) are another vector option deemed safer than lentiviruses for gene therapy because they are not designed to insert their cargo into a cell’s genome but rather remain as an episome in the nucleus of the transduced cells. However, although an AAV is considered a non-integrated vector, integrational events have been observed with a low frequency of 0.1–3% [53]. A phase 3 gene therapy trial for haemophilia using an AAV vector was halted by the FDA because a patient developed a liver tumour, although it was thought highly unlikely to be related to the gene therapy [72]. However, animal models have shown increased incidence of hepatocellular carcinoma after AAV liver gene transfer, which is alarming for potential vector-related tumourogenesis [73,74]. In addition, AAV type 2 has been associated with oncogenic insertional mutagenesis in humans with hepatocellular carcinomas through integrations in known cancer driver genes, such as CCNA2, TERT, CCNE1, TNFSF10 and KMT2B, leading to overexpression of the target genes [75].

Genetic engineering has massively changed the field of cell and gene therapy, allowing alternative, more precise technologies to achieve genetic manipulation, minimising the complexity and the risks of genotoxicity associated with viral vectors. Currently implemented methods of gene editing include non-viral delivery systems, such as CRISPR-Cas9, transposons, zinc-finger nucleases, transcription-activator-like effector nucleases and meganucleases [76]. These methods are being exploited in several research platforms to generate safer and more potent CAR T-cell therapies, while commercial products developed by harnessing gene-editing techniques (CRISPR-Cas9-based products) have been approved as gene therapy treatments for sickle cell and transfusion-dependent β-thalassemia patients [13,77,78]. However, genome editing is not always precise, resulting in off-target effects with mutations, genomic disruption and chromosome rearrangement with unknown biological and clinical significance [79,80,81]. More importantly, cases of secondary T-cell lymphoma have been reported after applying piggyBac, a transposon system for CAR gene delivery in the manufacturing of CAR T-cells. The lymphomagenesis was related to high transgene insertion copy numbers, but it was thought that the widespread genomic copy number variations found within the genome of lymphoma cells was the dominant driver of carcinogenesis [30,82]. Notably, in this phase 1 trial, it was found that 7% of the transgene integration sites occurred within cancer-related genes, but these were not different than those previously reported with similar gene delivery methods. This trial was suspended by the FDA after the occurrence of these CAR-positive T-cell lymphoma cases [30]. The high insertion copy numbers are in line with the importance of the vector copy number in the use of viral vectors. The vector copy number reflects the number of integrated transgene copies within a CAR T-cell product and remains one of the FDA’s quality and safety requirements (less than five vector copies per transduced cell), as it correlates with the product potency, but it may also reflect the oncogenic potential, as greater copy numbers are associated with a greater risk of random vector integration into the host genome [83].

Base editing is a novel technology editing only targeted single DNA bases rather than removing, adding or altering sections of the DNA sequence; therefore, it is deemed safer in terms of genotoxicity. However, despite being more accurate, base editing has been found to trigger mutations at random sites in the genome beyond the target site [84,85]. Ongoing research may lead to the identification of genomic safe harbours in parallel with the development of new strategies that target the insertion of the CAR construct to these specific loci within the genome, avoiding the risk of oncogenesis [86].

## 4. A Proposed Strategy for Future Research Aiming to Mitigate the Risk of Secondary Malignancies

Due to the theoretical risk of insertional mutagenesis, all CAR T-cell-treated patients have been under long-term surveillance. Therefore, the FDA announcement of secondary T-cell malignancies after CAR T-cell therapies came as a surprise to the scientific community. The confirmation of these allegations has raised concerns about the future of CAR T-cell therapies and cell and gene therapies in general, as different genetically manipulated products are underway to be applied in the clinical setting for various disease types, both malignant and non-malignant. The exact causes driving the development of secondary malignancies after CAR T-cell therapies are unclear. Regarding the T-cell malignancies, none of the 22 reported cases of secondary T-cell malignancies have been explicitly related to vector-mediated insertional oncogenicity. This number could be an underestimation of the true incidence, as more than 34,400 patients have been treated with commercial CAR T-cell therapies and only 8000 cases have been officially reported with adverse events [24]. Apparently, the decoding of the triggering mechanisms of secondary T-cell and other malignancies requires the wider study of further potentially unreported cases. This is a laborious and lengthy follow-up process and is consistent with the advance of genetic engineering.

**Figure 2:**
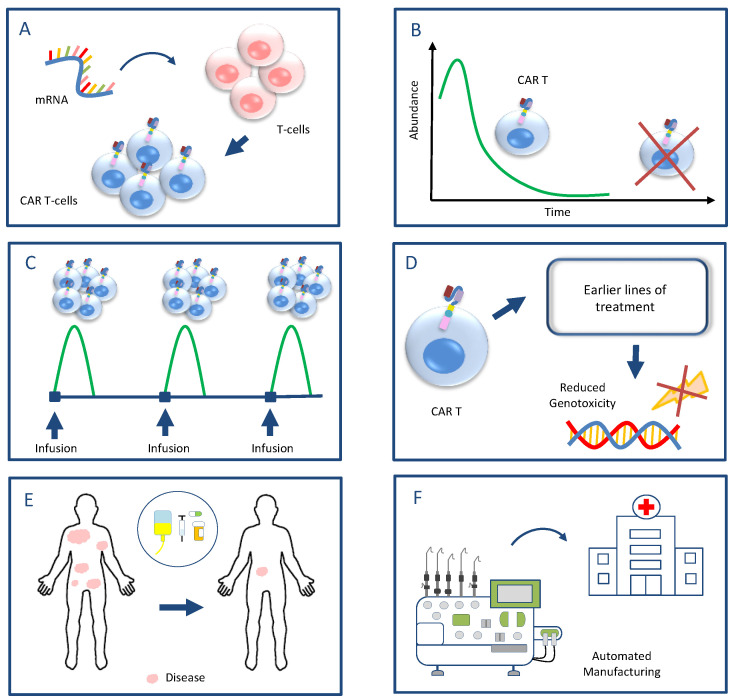
A proposed strategy for future research aiming to mitigate the risks of developing secondary malignancies after CAR T-cell therapies: (**A**) harnessing of mRNA technologies for the CAR introduction to avoid genotoxicity caused by viral vectors or gene editing; (**B**) short CAR T-cell persistence to diminish immune dysregulation, which may drive clonal evolution and the development of secondary malignancies; (**C**) infusion of high cell doses with defined cell compositions in fractions, which can increase the likelihood of early disease eradication, making unnecessary CAR T-cell persistence; (**D**) move of CAR T-cell therapies to earlier lines of treatment to avoid accumulated genotoxicity caused by previous intense treatments; (**E**) effective control of disease burden prior to infusion to reduce the detrimental effects of subsequent inflammation; (**F**) promotion of automated in-house CAR T-cell manufacturing to reduce the production time and the risk of disease progression with high tumour burden.

Based on the reported scientific evidence characterising the nascent and unclear current landscape of secondary malignancies after CAR T-cell infusion and the potential mechanisms of their development, a composite set of six proposals is presented for future research. If this suggestive strategy is confirmed, it can potentially contribute to mitigating or preventing the risks of developing secondary malignancies either by avoiding genotoxicity or reducing CAR T-associated inflammation and the immunosuppressive microenvironment (Figure 2):

A. Exploitation of mRNA technologies instead of viral vectors or gene-editing techniques for the CAR introduction to avoid random transgene integration into the host cells and subsequent genotoxicity. mRNA technologies have been proven very efficient in translating the CAR protein. Preliminary results from experimental CAR T-cell platforms harnessing mRNA techniques have shown promising safety and efficacy in eliminating tumour cells [87,88] (Figure 2A).

B. Short CAR T-cell persistence to reduce the period of overt and subclinical inflammation and immune dysregulation, which may drive the occurrence of abnormal clonal haematopoiesis, the clonal evolution of already pre-existing aberrant clones to malignancies and the development of secondary lymphomas (Figure 2B).

C. Infusion of high cell doses with defined cell compositions in fractions, as it seems that doses with high cell numbers have the ability to randomly eliminate malignancies early after infusion and offer a cure [89,90]. In this perspective, long-term CAR T-cell persistence does not seem to be necessary but rather augments the risk of further adverse events and toxicities (Figure 2C).

D. Move of CAR T-cell therapies to earlier lines of treatment to avoid the accumulation of chemotherapy or radiotherapy-related genotoxicity and the development of pre-malignant clones (Figure 2D).

E. Effective control of disease burden prior to infusion to reduce the subsequent generation of excessive CAR T-cell-related inflammation and the potential detrimental effects of inflammation (Figure 2E).

F. Promotion of automated in-house CAR T-cell manufacturing to reduce production time and complexity as a measure to prevent disease progression and an increase in the disease burden prior to CAR T-cell infusion (Figure 2F).

## 5. Conclusions

The announcement on 28 November 2023 by the FDA concerning the occurrence of rare secondary T-malignancies after CAR T-cell therapies should in no way act as a brake on the further clinical use of CAR T-cells. Given the expanding indications of treating diseases with the CAR T-cell technology, this type of cell therapy seems to enclose the potential to be converted into the “cellular penicillin” of the 21st century, a lifesaving and survival-prolonging treatment. Therefore, it seems imperative to render CAR T-cell therapies a safer treatment modality. The abovementioned strategy can pave the way for extensive research and bridge the existing therapeutic gap in CAR T-cell therapies in order to turn CAR T-cell therapies into a treatment with a higher curative potential and sparing toxic or even life-threatening side effects.

## Figures and Tables

**Table 1:** Summary of the currently approved CAR T-cell products by the FDA for commercial application.

CAR T-Cell Product	Brand Name	Company	Disease Indications (R/R)	Year Initially Approved	Target Antigen	Costimulatory Domain	Viral Vector
Tisagenlecleucel	Kymriah	Novartis	B-ALL (≤25 years) LBCL FL	2017	CD19	4-1BB	Lentiviral
Axicabtagene ciloleucel	Yescarta	Kite	LBCL FL	2017	CD19	CD28	Retroviral
Brexucabtagene autoleucel	Tecartus	Kite	B-ALL (>25 years) MCL	2020	CD19	CD28	Retroviral
Lisocabtagene maraleucel	Breyanzi	Juno/BMS	LBCL MCL FL CLL/SLL (AA)	2021	CD19	4-1BB	Lentiviral
Idecabtagene vicleucel	Abecma	Celgene/BMS	MM	2021	BCMA	4-1BB	Lentiviral
Ciltacabtagene autoleucel	Carvykti	Legend/J&J	MM	2022	BCMA	4-1BB	Lentiviral

Abbreviations: AA, accelerated approval; B-ALL, B-cell acute lymphoblastic leukaemia; BCMA, B-cell maturation antigen; BMS, Bristol-Myers Squibb; CAR, chimeric antigen receptor; CLL, chronic lymphocytic leukaemia; FDA, Food and Drug Administration; FL, follicular lymphoma; J&J, Johnson & Johnson; LBCL, large B-cell lymphoma; MCL, mantle cell lymphoma; MM, multiple myeloma; R/R, relapsed/refractory; SLL, small lymphocytic lymphoma.
